# Screening for Plant Volatile Emissions with Allelopathic Activity and the Identification of L-Fenchone and 1,8-Cineole from Star Anise (*Illicium verum*) Leaves

**DOI:** 10.3390/plants8110457

**Published:** 2019-10-28

**Authors:** Gaowa Kang, Maryia Mishyna, Kwame Sarpong Appiah, Masaaki Yamada, Akihito Takano, Valery Prokhorov, Yoshiharu Fujii

**Affiliations:** 1 Department of Biological Production Science, United Graduate School of Agriculture, Tokyo University of Agriculture and Technology, Fuchu, Tokyo 183-8509, Japan; kanggaowa1111@yahoo.co.jp (G.K.); ksappiah90@gmail.com (K.S.A.); 2 School of Food Science and Biotechnology, Zhejiang Gongshang University, 18, Xuezheng Street Hangzhou, Zhejiang 310018, China; maryia.mishyna@gmail.com; 3 Department of International Environmental and Agricultural Science, Faculty of Agriculture, Tokyo University of Agriculture and Technology, Tokyo 183-8509, Japan; masaakiy@cc.tuat.ac.jp; 4 Faculty of Pharmaceutical Sciences, Showa Pharmaceutical University, Machida, Tokyo 194-8543, Japan; takano@ac.shoyaku.ac.jp; 5 Laboratory of Plant Growth and Development, Institute of Experimental Botany, National Academy of Sciences of Belarus, 220 072 Minsk, Belarus; prohoroff1960@mail.ru

**Keywords:** allelopathy, camphene, *Illicium verum*, L-fenchone, star anise, volatile, 1,8-cineole, β-pinene

## Abstract

One hundred and thirty-nine medicinal plant species were screened for their allelopathic activity through volatile emissions using *Lactuca sativa* as a test plant. Volatile emissions from the leaves of star anise (*Illicium verum*) showed the highest inhibition (100%) on the radicle and hypocotyl growth. Using headspace gas collection and gas chromatography-mass spectrometry (GC-MS), seven major volatile compounds from the leaves of star anise, including α-pinene, β-pinene, camphene, 1,8-cineole, D-limonene, camphor, and L-fenchone were detected. To determine volatile compounds that may contribute to the inhibitory activity of star anise, the allelopathic potential of individual volatiles from star anise was evaluated using the cotton swab bioassay. The EC_50_ was calculated for each of the seven identified compounds. L-fenchone showed the strongest growth inhibitory activity (EC_50_ is 1.0 ng/cm^3^ for radicle and hypocotyl growth of lettuce), followed by 1,8-cineole, and camphene. This is the first report that L-fenchone could be an important volatile allelochemical from the leaves of star anise. From the actual concentration of each volatile compound in headspace and EC_50_ value, we concluded that the four volatile compounds, including L-fenchone, 1,8-cineole, β-pinene, and camphene are the most important contributors to the volatile allelopathy of star anise.

## 1. Introduction

Allelopathy refers to any direct or indirect harmful or beneficial effect by an organism (mostly plants) on another species through the production of bioactive compounds that are released into the environment [1]. Besides, the importance of allelopathic interaction between plants in nature, screening, and identification of natural compounds with high allelopathic activity is one direction in the search for new natural herbicides that could augment current weed control approaches. Several natural compounds with allelopathic potential were discovered, including phenolic compounds, terpenoids, and alkaloids [2,3].

The identification of novel allelopathic compounds includes numerous screening tests, both in laboratory and field conditions, chemical screening [4,5], and evaluation of the most promising allelochemicals as a new plant growth regulator. Some of these allelochemicals may exert direct function by acting against pathogens or acting indirectly through the activation of the defense response of plants. Green leaf volatiles are synthesized via the hydroperoxide lyase (HPL) branch of the oxylipin pathway, which belongs to this category of defensive molecules [6,7]. Volatiles released from herbivore-infested plants can also mediate plant-plant interactions. This plant-plant interaction may induce the expression of emission of volatiles in healthy leaves on the same plant or of neighboring un-attacked plants, hence increasing their attractiveness to carnivores and decreasing their susceptibility herbivores [8]. Allelopathic potential of volatile compounds has been previously demonstrated for some green manure crops from Brassicaceae family (white, brown mustard, and black mustard, leafy turnip, rapeseed, garden cress) with the release of high levels of allyl isothiocyanate by mustard that can be optimal for allelopathic activity [9]. Volatiles compounds including 3-methyl-1-butanol, 3-hexen-1-ol, 2-heptanol, pentanal, 2-methylbutanal, 3-methylbutanal, and others were identified from amaranth residues and demonstrated the highest bioactivity toward test species suggesting their allelopathic importance [10]. Green leaf volatiles enhanced the attractant pheromone response of the boll weevil (*Anthonomus grandis*) *Scolytus multistriatus*, and *Ceratitis capitata* [11]. The treatment of plants with (Z)-3-hexenyl propionate resulted in stomatal closure, pathogenesis related (PR) gene induction, and enhanced resistance to the bacteria [12]. However, the potential of volatile allelochemicals is still poorly studied, although the use of volatile plant growth inhibitors may have practical importance in closed systems such as greenhouses.

Therefore, the aim of our study was *(i)* to screen 139 plant species to identify the most promising candidates with high allelopathic activity through volatile compounds, *(ii)* to evaluate the volatile profile of star anise leaves, and *(iii)* to determine the plant growth inhibitory activity of individual volatile compounds from star anise leaves.

## 2. Results and Discussion

### 2.1. Screening of Allelopathic Activity

**Figure 1:**
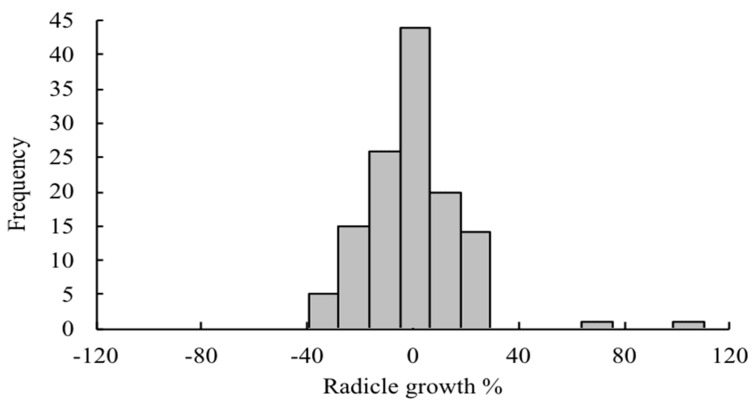
Distribution of plant species according to their radicle growth of lettuce.

**Figure 2:**
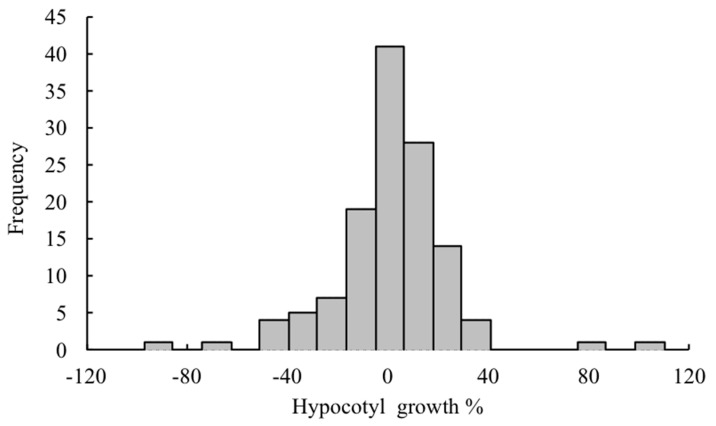
Distribution of plant species according to their hypocotyl growth of lettuce.

Allelopathic activity of volatile emissions from 139 plant species (Appendix A) using lettuce as a test plant was evaluated using the Dish pack method [13,14]. The top 30 plants with the highest allelopathic activity are presented in Table 1. Generally, 59% and 50% of the screened plants inhibited hypocotyl and radicle growth of lettuce seedlings respectively at different degrees (Figure 1 and Figure 2). Other plant species demonstrated either a lack of inhibitory activity or exhibited stimulatory activity up to 38.9% (*Epimedium sagittatum*) and 95.0% (*Pimenta racemosa*) for lettuce radicle and hypocotyl, respectively. The highest radicle and hypocotyl inhibition (100%) were observed for the volatile constituents of *Illicium verum* or star anise leaves. *I. verum* (Illiciaceae) is an aromatic evergreen tree distributed in North America, the West Indies, and Eastern Asia, and is known for the use of its fruits in traditional Chinese medicine and the food industry due to unique secondary metabolites, such as terpenoids, phenylpropanoids, lignans, and benzoquinones [15,16]. Both the leaves and fruits have a strong aroma with a distinctive licorice taste [17]. Star anise is known for its insecticidal activity [18], antifungal [19], and antimicrobial [20] properties. However, there is no information about the allelopathic properties of star anise through volatile emissions. Therefore, this plant was chosen as a candidate species for further identification of volatile compounds.

Several other plants with high plant growth inhibitory activity were also identified in this study. For example, the volatiles from *Crateva religiosa* or sacred garlic pear, suppressed the hypocotyl growth by 86.2%, followed by *Shorea robusta, Artabotrys uncinatus, Sinomenium acutum, Dendrobium* sp. (with inhibitory activity ranging from 34.4 to 30.3%). Regarding the inhibitory effect on radicle growth, *Argemone mexicana* or Mexican poppy suppressed 73.5% of lettuce radicle growth. Twenty-four other plant species showed radicle inhibition from 10.5% (*Zingiber officinale*, ginger) to 26.3% (*Derris malaccensis*). Generally, there was no significant correlation between radicle and hypocotyl growth, which can be due to differences in the mode of action of volatile compounds and their availability for lettuce seedlings. Although this study focused on the screening of plants with high plant growth inhibitory activity, several species showed a stimulatory effect, especially on hypocotyl growth. Some of these species include, but not limited to, *Pimenta racemose* (95% of stimulatory) from the myrtle family and *Citrus hystrix* (70.8%) or kaffir lime.

**Figure 3:**
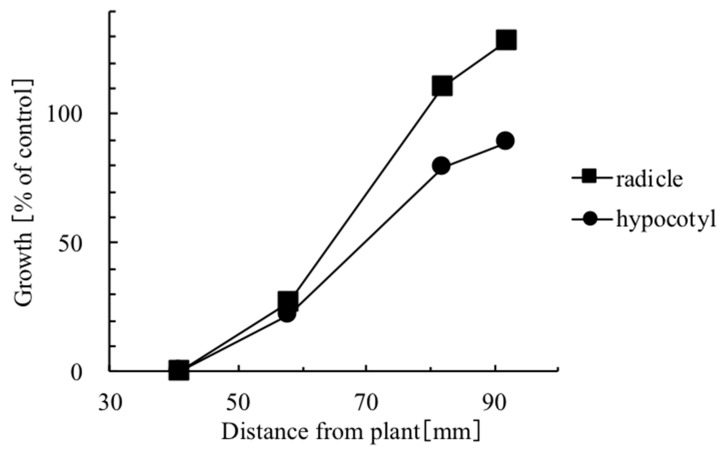
Effect of leaf volatiles from star anise on radicle and hypocotyl growth [%] of lettuce seedlings as a function of distance from plant material using the Dish Pack method.

While the data presented in Table 1 were obtained from wells that were located 41 mm from the plant source, the distance from the source of volatiles had a significant effect on the observed inhibitory activity. In this regard, Figure 3 shows that the growth inhibitory effect of volatiles from star anise decreased as a function of a distance from a well for both radicle and hypocotyl.

### 2.2. The GC-MS Analysis of Volatiles Constituents from the Leaves of Star Anise

Seven major compounds (α-pinene (5.2%), β-pinene (13.4%), camphene (7.8%), D-limonene (7.2%), 1,8-cineole (17.9%), L-fenchone (6.9%), and camphor (7.2%) were detected in the headspace of star anise by GC-MS. These identified volatile constituents differed from previously reported volatile compositions because we calculated headspace volatile from the leaf of *I. verum*. *Trans*-anethole is an important component of aromatic volatile in *I. verum* [21], but this compound is only found in the fruit and not in the leaves. Previous analysis of chemical components of star anise fruit using hydro distillation–headspace solvent microextraction followed by GC-MS revealed 49 compounds, mainly including *trans*-anethole (81.4%), limonene (6.50%), chavicol (2.10%), and anisaldehyde (1.81%) [22]. The major volatile components of *I. verum* fruit oil in a study by Huang et al. [17] were *trans*-anethole (87.7%) and 4-allyl anisole (6.7%). In order to evaluate the antifungal activity of essential oil of star anise, GC-MS analysis was conducted, and *trans*-anethole (89.5%), 2-(1-cyclopentenyl)-furan (0.9%) and *cis*-anethole (0.7%) were found to be the main volatiles among the 22 identified compounds [19]. However, the identified volatile compounds in star anise essential oil differed from those identified in leaf oil of *I. lanceolatum* and mainly contained β-linalool (16.2%), elemicin (14.9%), and cineole (14.8%) [17]. Oil from the three *Illicium* species was obtained by hydrodistillation and analyzed by GC (FID) and GC-MS. As a result, the difference between species was observed, i.e. major constituents of *I. majus* were aromadendrene (13.0%), cuparene (8.2%), 1,8-cineole (8.1%) and calamenene (7.8%), *I. micranthun* - 1,8-cineole (8.4%), linalool (7.7%), (E)-nerolidol (7.6%) and sabinene (7.1%), *I. tsaii* - (E)-nerolidol (15.5%), β-caryophyllene (8.1%), β-cedrene (6.5%), 1,8-cineole (6.3%) and calamenene (6.3%) [23]. Neto et al. [24] demonstrated that the vapour phase of star anise essential oil had fungicidal activity against *Penicillium expansum*. *Trans*-Anethole, a major component of star anise essential oil, was found to be an effective repellent and oviposition deterrent [25].

### 2.3. Evaluation of EC_50_ of Volatiles from Star Anise Volatiles

**Figure 4:**
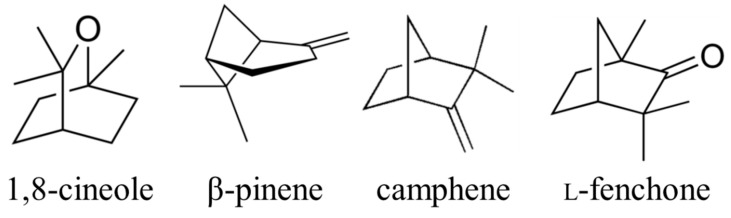
Structure of major volatile compounds from star anise leaves.

Inhibitory activity of the authentic volatile compounds varied from D-limenone (EC_50_ is 105.7 ng/cm^3^ and 24 5 ng/cm^3^ for hypocotyl and radicle, respectively, less the inhibitor) to L-fenchone (EC_50_ is 1.0 ng/cm^3^ for radicle and hypocotyl, most potent inhibitory activity). Similar to this study, volatile terpenes, including camphor, 1,8-cineole, α-pinene, and β-pinene, were identified from the invasive perennial weed mugwort (*Artemisia vulgaris*), and their potential role in mugwort establishment and proliferation in introduced habitats was suggested to be as a result of their phytotoxicity [26]. The determination of EC_50_ in the headspace (Table 2) of the seven compounds showed that L-fenchone was the most potent plant growth inhibitor (EC_50_ is 1.0 ng/cm^3^ for both radicle and hypocotyl), followed by 1,8-cineole and camphene. Kaur et al. [27] demonstrated that the volatiles from the essential oil of *Eucalyptus tereticornis*, including α-pinene (32.5%) and 1,8-cineole (22.4%), significantly suppressed early seedling growth and seedling vigour of *Amaranthus viridis*. 1,8-Cineole is known to be a potent plant growth regulator and can inhibit mitosis, which leads to growth abnormities, inhibits respiration of isolated mitochondria, and aspartate synthase [28]. In fennel seeds, L-fenchone is well known to be present in sufficient amounts, but L-fenchone was never reported as potent plant growth inhibitors. This is the first report that L-fenchone could be an important volatile allelochemical from the leaves of the star anise. From the actual concentration of each volatile compound in the headspace and EC_50_ value, we concluded that four volatile compounds, 1,8-cineole, β-pinene, camphene, and L-fenchone (Figure 4) were the most important contribution for plant growth inhibitory activity in the headspace of star anise.

The cotton swab method, following GC-MS analysis was previously successfully applied for the determination of EC_50_ of radicle and hypocotyl growth of lettuce seedlings by 1-decyne in the vapor phase and was found at the concentration of 0.5 ng/mL [29] and by safranal—1.2 µg/L (ppb) [30]. Additionally, previous results also demonstrated that octyl acetate, a major volatile from *H. sosnowskyi* fruits, had lower EC_50_ for radicle and hypocotyl growth (64 and 57 ng/cm^3^, respectively), than the predominant octanal (EC_50_ is 20 and 9 ng/cm^3^ respectively), however, octanal was suggested to be the major contributor to its allelopathic activity based on total activity estimation [14].

## 3. Materials and Methods

### 3.1. Plant Material

Plant materials (leaves) were collected from 139 plant species in the Botanical Garden of Showa Pharmaceutical University, Tokyo (Japan), from May to June 2013. All samples were dried in an oven at 60 °C for 4 h and then stored in paper bags placed in plastic bags in a refrigerator (4 °C) before their use.

### 3.2. Dish Pack Method

**Figure 5:**
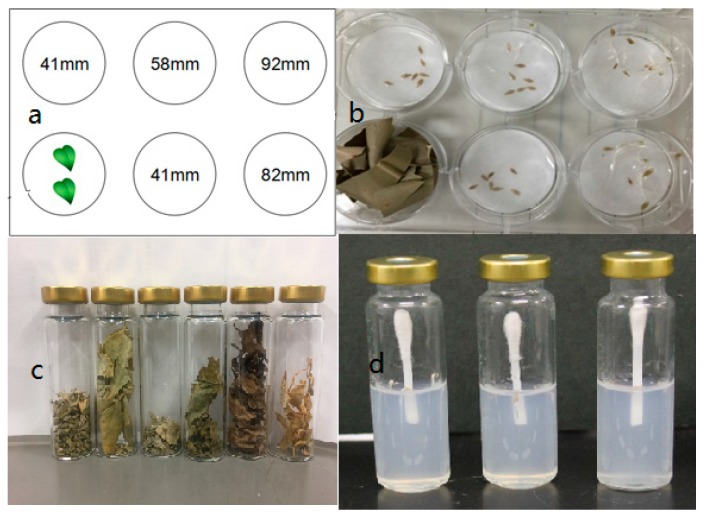
Testing the effect of leave volatiles from star anise on radicle and hypocotyl growth (%) of lettuce seedlings as a function of distance from plant material using the Dish Pack method (**a**,**b**), samples for GC-MS analysis (**c**), and cotton swab method (**d**).

The dish pack method [13] was used to determine the allelopathic activity of naturally emitted volatile compounds of test plant materials (Figure 5). Briefly, 2 g of dried material was placed in one of the 6-well multi-dish plastic plate (3.5 cm d., Nunc Company). The distances from the well where the sample was placed (source well) to the center of other wells were 41, 58, 82 and 92 mm (Figure 5a). In each of the other 5 wells, the filter paper was placed, and 0.7 mL of distilled water was added. Then, 7 seeds of *Lactuca sativa*, var. Great Lakes 366 (Takii seed Co., Japan) were placed on top. The plastic plates were sealed tightly and incubated for 3 days at 22 °C under dark conditions. A multi-dish plastic plate with a blank source well was used as the control treatment. The lengths of lettuce radicle and hypocotyl were measured, and the allelopathic activity was expressed as a percentage of radicle or hypocotyl inhibition at wells located 41 mm from the plant source.

### 3.3. Headspace Gas Chromatography-Mass Spectrometry

Plant material (1 g) was placed into a 20 mL sealed glass vial (GRACE, Japan) and incubated at 60 °C for 1 h. Then, headspace gas (200 μL) was collected using a 1000 μL micro-syringe (MS-GAN100, Ito Corporation, Tokyo, Japan), and injected into a gas chromatography-mass spectrometry set-up (GC-MS-QP 2010 Plus system, Shimadzu, Japan) using an EQUITY-5 column (0.25 mm × 30 m × 0.25 μm, Supelco) [14]. Helium gas was used as a carrier with a total flow rate of 29 mL/min. The injection temperature was 200 °C with a column head pressure of 61.3 kPa. The oven temperature was increased at a rate of 10 °C/min to 200 °C from 60 °C and kept constant for 30 min at the end. Mass spectra were recorded at 70 eV and compared with an in-house mass spectral library (NIST and Wiley). The samples analyzed using the headspace GC-MS were the leaves of the *Illicium verum* and the volatile compounds, including α-pinene, β-pinene, camphene, 1,8-cineole, D-limonene, camphor, and L-fenchone.

### 3.4. Cotton Swab Method

The cotton swab method [14] was used to evaluate the plant growth inhibitory activity of the leaves of *I. verum* and authentic volatile compounds 1,8-cineole, beta-pinene, camphene, D-limonene, α-pinene, camphor, and L-fenchone, which were identified by GC-MS analysis as major volatile compounds. Briefly, 10 mL of 0.75% agar solution was added to a 20 mL glass vial, and after agar solidification, 7 seeds of lettuce were placed into each vial. A half of double-tipped cotton [14] was vertically inserted into the agar, and an appropriate amount (0.1, 0.2, and 0.3 µL) of the authentic compound was added on the cotton swab. The concentrations of the compounds were 0.001, 0.01, 0.1, and 1 ppm. The glass vial was closed by a pressure cap and incubated for 3 days at 22 °C. The length of the radicle and hypocotyl of the lettuce seedlings were measured, and the inhibition of the radicle and hypocotyl of the lettuce seedlings was plotted against the applied amount of an authentic compound.

### 3.5. EC_50_ of Authentic Volatile Compounds

The EC_50_ of authentic volatiles compounds α-pinene, β-pinene, camphene, 1,8-cineole, D-limonene, camphor, and L-fenchone were determined using the cotton swab method. The GC-MS analysis of headspace to determine the actual concentration of volatiles in the leaves of star anise was done as described above. The EC_50_ value was expressed in ng/cm^3^.

## 4. Conclusions

The screening of 139 plants from Japan revealed several plants with potential volatile allelopathic activity (inhibitory or stimulatory). The information obtained about plant growth inhibitory or stimulatory activity can be used as a benchmark for the studies on the suppression of weeds or use as plant growth regulators. In this study, the volatile compounds from star anise leaves inhibited (100%) the radicle and hypocotyl growth of lettuce seedlings. α-pinene, β-pinene, camphene, 1,8-cineole, D-limonene, camphor, and L-fenchone were identified as the main volatile compounds. These compounds were previously reported as constituents of several essential oils with potent bioactivity. However, our results demonstrated that L-fenchone, 1,8-cineole, β-pinene, and camphene could be significant contributors to the volatile allelopathy of star anise leaves.

## Figures and Tables

**Table 1:** Top 30 plant with the most potent inhibitory activity of radicle and hypocotyl growth of lettuce (*L. sativa*) seedlings (% compared to control) through volatiles.

Name	Family	H (%)	Criteria	R (%)	Criteria
*Illicium verum* Hook.f	Schisandraceae	0.0	****	0.0	****
*Crateva religiosa* G.Forst.	Capparaceae	13.8	****	105.0	
*Shorea robusta* C.F.Gaertn.	Dipterocarpaceae	65.6	***	109.0	
*Artabotrys uncinatus* (Lam.) Merr.	Annonaceae	68.2	***	101.0	
*Sinomenium acutum* (Thunb.) Rehder et E.H.Wilson	Menispermaceae	68.8	***	101.0	
*Cinnamomum cassia* (L.) J.Presl	Lauraceae	68.9	***	97.0	
*Dendrobium* sp.	Orchidaceae	69.7	***	95.0	
*Ricinus communis* L.	Euphorbiaceae	75.3	**	93.0	
*Atractylodes chinensis* (Bunge) Koidz.	Asteraceae	77.2	**	91.0	
*Cinnamomum burmannii* (Nees & T.Nees) Blum	Lauraceae	77.3	**	87.0	*
*Tabebuia chrysotricha* (Mart. ex DC.) Standl.	Bignoniaceae	77.3	**	89.0	*
*Piper longum* L.	Piperaceae	78.2	**	85.0	*
*Terminalia bellirica* (Gaertn.) Roxb.	Combretaceae	78.5	**	83.0	*
*Clivia nobilis* Lindl.	Amaryllidaceae	78.6	**	107.1	
*Tinospora tuberculata* (Lam.) Beumée ex K.Heyne	Menispermaceae	79.5	*	94.3	
*Malpighia glabra* L.	Malpighiaceae	79.5	**	109.8	
*Arctium lappa* L.	Asteraceae	80.3	*	75.8	**
*Podophyllum peltatum* L.	Berberidaceae	80.7	*	84.5	*
*Ceiba pentandra* (L.) Gaertn.	Malvaceae	81.8	*	100.6	
*Santalum album* L.	Santalaceae	81.8	*	115.2	
*Acacia catechu* (L.f.) Willd.	Fabaceae	82.7	*	94.0	
*Polygala senega* L. var. *latifolia* Torr. et A.Gray	Polygalaceae	83.5	*	108.6	
*Tectona grandis* L.f.	Lamiaceae	83.9	*	105.0	
*Valeriana fauriei* Briq.	Caprifoliaceae	84.7	*	116.6	
*Derris elliptica* (Wall.) Benth.	Fabaceae	85.1	*	115.1	
*Celosia argentea* L.	Amaranthaceae	85.5	*	90.4	
*Ficus religiosa* L.	Moraceae	85.6	*	88.3	*
*Croton sublyratus* Kurz	Euphorbiaceae	86.0	*	95.8	
*Eucommia ulmoides* Oliv.	Eucommiaceae	87.2	*	108.1	
*Achras sapota* L.	Sapotaceae	87.5	*	110.1	
Mean (M)		98.0		100.7	
Standard Deviation (SD)		18.4		21.5	
M-0.5 SD		88.8	*	89.9	*
M-1.0 SD		79.6	**	79.2	**
M-1.5 SD		70.4	***	68.5	***
M-2.0 SD		61.2	****	57.7	****

More [*] indicate stronger plant growth inhibitory activity. H: Hypocotyl (% of control), R: Radicle (% of control).

**Table 2:** Inhibitory activity (EC_50_) of the seven major compounds detected in the headspace of star anise on radicle and hypocotyl growth of lettuce seedlings.

RT (min)	Name of Compounds	% of Compound	EC_50_ [ng/cm^3^]	
			Radicle	Hypocotyl
9.43	α–pinene	5.2	19.7 ± 8.2	16.2 ± 11.3
9.78	camphene	7.8	4.6 ± 0.2	5.7 ± 0.7
10.47	β-pinene	13.4	7.7 ± 3.5	6.5 ± 1.1
11.61	D-limonene	7.2	105.7 ± 67.6	24.0 ± 10.2
11.66	1,8-cineole	17.9	3.7 ± 0.6	3.8 ± 1.6
12.80	L-fenchone	6.9	1.0 ± 0.2	1.0 ± 0.3
13.86	camphor	7.2	13.0 ± 3.6	7.6 ± 1.5

RT: Retention time, Data are the mean of three replications ± standard deviation.

## Supplementary Materials

Supplementary File 1

## Appendix A

Potential allelopathic activity on the radicle and hypocotyl growth of lettuce (*L. sativa*) seedlings through volatiles constituents of tested plant species.

## References

[1] Rice E.L. (1984). Allelopathy.

[2] Macias F.A. (1994). Allelopathy in the search for natural herbicide models. Am. Chem. Soc..

[3] Macías F.A., Mejías F.J., Molinillo J.M. (2019). Recent advances in allelopathy for weed control: From knowledge to applications. Pest. Manag. Sci..

[4] Fujii Y., Parvez S.S., Parvez M.M., Ohmae Y., Iida O. (2003). Screening of 239 medicinal plant species for allelopathic activity using the sandwich method. Weed Biol. Manag..

[5] Wu H., Pratley J., Lemerle D., Haig T., An M. (2001). Screening methods for the evaluation of crop allelopathic potential. Bot. Rev..

[6] Howe G.A., Schilmiller A.L. (2002). Oxylipin metabolism in response to stress. Curr. Opin. Plant Biol..

[7] Dudareva N., Negre F., Nagegowda D.A., Orlova I. (2006). Plant Volatiles: Recent Advances and Future Perspectives. Crit. Rev. Plant. Sci..

[8] Ruther J., Kleier S. (2005). Plant-plant signaling: Ethylene synergizes volatile emission in *Zea mays* induced by exposure to (Z)-3-hexen-1-ol. J. Chem. Ecol..

[9] Vaughn S.F., Boydston R.A. (1997). Volatile allelochemicals released by crucifer green manures. J. Chem. Ecol..

[10] Connick W.J., Bradow J.M., Legendre M.G. (1989). Identification and bioactivity of volatile allelochemicals from amaranth residues. J. Agric. Food Chem..

[11] Visser J.H., Van Straten S., Maarse H. (1979). Isolation and identification of volatiles in the foliage of potato, *Solanum tuberosum*, a host plant of the Colorado beetle, *Leptinotarsa decemlineata*. J. Chem. Ecol.

[12] López-Gresa M.P., Payá C., Ozáez M., Rodrigo I., Conejero V., Klee H., Bellés J.M., Lisón P. (2018). A New Role for Green Leaf Volatile Esters in Tomato Stomatal Defense Against *Pseudomonas syringe* pv. tomato. Front. Plant Sci..

[13] Fujii Y., Matsuyama M., Hiradate S., Shimozawa H. (2005). Dish pack method: A new bioassay for volatile allelopathy. Thymus.

[14] Mishyna M., Laman N., Prokhorov V., Maninang J.S., Fujii Y. (2015). Identification of octanal as plant growth inhibitory volatile compound released from *Heracleum sosnowskyi* fruit. NPC Nat. Prod. Commun..

[15] Wang G.-W., Hu W.-T., Huang B.-K., Qin L.-P. (2011). *Illicium verum*: A review on its botany, traditional use, chemistry and pharmacology. J. Ethnopharmacol..

[16] Liu Y.-N., Su X.-H., Huo C.-H., Zhang X.-P., Shi Q.-W., Gu Y.-C. (2009). Chemical constituents of plants from the genus *Illicium*. Chem. Biodivers..

[17] Huang B., Liang J., Wang G., Qin L. (2012). Comparison of the Volatile Components of *Illicium verum* and *I. lanceolatum* from East China. J. Essent. Oil Bear. Plants.

[18] Szczepanik M., Szumny A. (2011). Insecticidal activity of star anise (*Illicum verum* Hook. F.) fruits extracts against lesser mealworm, *Alphitobius diaperinus* Panzer (Coleoptera: *Tenebrionidae*). Allelopath. J..

[19] Huang Y., Zhao J., Zhou L., Wang J., Gong Y., Chen X., Guo Z., Wang Q., Jiang W. (2010). Antifungal activity of the essential oil of *Illicium verum* fruit and its main component trans-anethole. Molecules.

[20] De M., De A.K., Sen P., Banerjee A.B. (2002). Antimicrobial properties of star anise (*Illicium verum* Hook F.). Phytother. Res..

[21] Zhang Y., Ji H., Yu J. (2018). Aromatic constituents and their changes of *Illicium verum* processed by different heating methods. Ind. Crops Prod..

[22] Gholivand M.B., Rahimi-Nasrabadi M., Chalabi H. (2009). Determination of essential oil components of star anise (*Illicium verum*) using simultaneous hydrodistillation—static headspace liquid-phase microextraction—Gas chromatography mass spectrometry. Anal. Lett..

[23] Qinh N.B., Dai D.N., Than B.V., Dung V.T., Hang V.T.T., Ogunwande I.A. (2016). Volatile constituents of three *Illicium* plants. Rec. Nat. Prod..

[24] Da Rocha Neto A.C., Navarro B.B., Canton L., Maraschin M., Di Piero R.M. (2019). Antifungal activity of palmarosa (*Cymbopogon martinii*), tea tree (*Melaleuca alternifolia*) and star anise (*Illicium verum*) essential oils against *Penicillium expansum* and their mechanisms of action. LWT.

[25] Chiluwal K., Kim J., Bae S., Park C.G. (2017). Essential oils from selected wooden species and their major components as repellents and oviposition deterrents of *Callosobruchus chinensis* (L.). J. Asia. Pac. Entomol..

[26] Barney J.N., Hay A.G., Weston L.A. (2005). Isolation and characterization of allelopathic volatiles from mugwort (*Artemisia vulgaris*). J. Chem. Ecol..

[27] Kaur S., Singh H.P., Batish D.R., Kohli R.K. (2011). Chemical characterization and allelopathic potential of volatile oil of *Eucalyptus tereticornis* against *Amaranthus viridis*. J. Plant Interact..

[28] Duke S., Oliva A., Macias F., Galindo J., Molinillo J., Cutler H. (2004). Mode of action of phytotoxic terpenoids. Allelopathy, Chemistry and Mode of Action of Allelochemicals.

[29] Elmadni H.S.A.M., Mishyna M., Fujii Y. (2019). Identification of 1-decyne as a new volatile allelochemical in baobab (*Adansonia digitata*) from Sudan. Afr. J. Agric. Res..

[30] Mardani H., Sekine T., Azizi M., Mishyna M., Fujii Y. (2015). Identification of safranal as the main allelochemical from saffron (*Crocus sativus*). Nat. Prod. Commun..

